# Exploiting proteomic data for genome annotation and gene model validation in *Aspergillus niger*

**DOI:** 10.1186/1471-2164-10-61

**Published:** 2009-02-04

**Authors:** James C Wright, Deana Sugden, Sue Francis-McIntyre, Isabel Riba-Garcia, Simon J Gaskell, Igor V Grigoriev, Scott E Baker, Robert J Beynon, Simon J Hubbard

**Affiliations:** 1 Dept Veterinary Preclinical Sciences, University of Liverpool, Crown Street, Liverpool, L69 7ZJ, UK; 2 Faculty of Life Sciences, University of Manchester, Manchester, M13 9PT, UK; 3 MBCMS, MIB, University of Manchester, Manchester, M13 9PT, UK; 4 DOE Joint Genome Institute, 2800 Mitchell Drive, Walnut Creek, CA 94598, USA; 5 Pacific Northwest National Laboratory, 902 Battelle Blvd, Richland, WA 99354, USA

## Abstract

**Background:**

Proteomic data is a potentially rich, but arguably unexploited, data source for genome annotation. Peptide identifications from tandem mass spectrometry provide *prima facie *evidence for gene predictions and can discriminate over a set of candidate gene models. Here we apply this to the recently sequenced *Aspergillus niger *fungal genome from the Joint Genome Institutes (JGI) and another predicted protein set from another *A.niger *sequence. Tandem mass spectra (MS/MS) were acquired from 1d gel electrophoresis bands and searched against all available gene models using Average Peptide Scoring (APS) and reverse database searching to produce confident identifications at an acceptable false discovery rate (FDR).

**Results:**

405 identified peptide sequences were mapped to 214 different *A.niger *genomic *loci *to which 4093 predicted gene models clustered, 2872 of which contained the mapped peptides. Interestingly, 13 (6%) of these *loci *either had no preferred predicted gene model or the genome annotators' chosen "best" model for that genomic locus was not found to be the most parsimonious match to the identified peptides. The peptides identified also boosted confidence in predicted gene structures spanning 54 introns from different gene models.

**Conclusion:**

This work highlights the potential of integrating experimental proteomics data into genomic annotation pipelines much as expressed sequence tag (EST) data has been. A comparison of the published genome from another strain of *A.niger *sequenced by DSM showed that a number of the gene models or proteins with proteomics evidence did not occur in both genomes, further highlighting the utility of the method.

## Background

Post genomic research and systems biology have greatly expanded our knowledge and understanding of biological processes, fuelled by the growth in sequenced genomes and accompanying technological developments. These techniques, such as microarray-based transcriptomics and proteomics, are reliant on the high quality annotation of newly sequenced genomes. Indeed, this heavy dependency on a sequenced genome or cDNA library can often be limiting in the scope of studies, particularly for non model organisms [1]. However, functional genomics experiments on sequenced organisms can also play an important role in defining or re-evaluating the genome sequenced on which they are based. Experimental data can be fed back into the genome to help demonstrate the validity or otherwise of the original gene structure predictions or to assist the annotation of new genomes.

Many genome sequencing projects use a range of *in silico *prediction methods to generate a large, and sometimes highly redundant, set of possible open reading frames (ORFs) and gene structure models. A good example is the pipeline employed by the widely-used Ensembl genome browser [2]. Here, a combination of EST, cDNA, orthology and statistical data are used to derive gene sets which are reconciled to produce a final set of high quality predicted genes. A further example is provided by recent fungal genomes sequenced at the US DOE Joint Genome Institute (JGI) whereby a large set of gene models are produced, typically with several candidates for each locus. Further analyses reduce this to a smaller filtered set of "best" gene predictions *via *a second layer of bioinformatic methods, manual annotation and the use of experimental data. It is one such example, that of *Aspergillus niger*, which forms the basis for this study. *A. niger *is a common ascomycete fungus that acts as an opportunistic human pathogen, however, it is generally more commonly known for its use in industrial biotechnological applications such as the production of citric acid [3]. We wished to apply mass spectrometry-based proteomics on *A. niger *as an exemplar system with which to test the utility of proteomics to refine and process a recently sequenced and annotated genome and produce an even higher quality gene set. There have already been several studies of the proteomics of filamentous fungi, now that there are several complete genome sequences, and this technique is being widely applied to understand fungal biology [4].

Although cDNA and oligonucleotides arrays can demonstrate that a predicted gene is expressed [5,6] and tiling arrays can define exon-intron structure with exquisite accuracy [7], they still focus on the un-translated mRNA. Proteomics provides a higher level confirmation of gene expression and is beginning to be used in genome annotation [8-10]. Mass spectrometry (MS) is an effective and fast method for identifying proteins from their constituent peptides and recent developments support much higher coverage of the commonly expressed proteome [11-13]. For example, Aerbersold and colleagues demonstrated how the PeptideAtlas database could be exploited to map many thousands of peptides back on to the human proteome [14,15]. Similarly, cDNA/EST data and mass spectrometry experiments have been used to identify novel ORFs and splice variants. Peptide identifications in expressed sequence tags (ESTs) [16] or expressed peptide tags (ePSTs) [17,18] were matched back to the genomic scaffolds, thereby identifying or validating real ORFs. Experimental proteomic data can therefore help with the prediction and validation of predicted gene structure and there are a growing number of examples which have helped annotate translational start sites, exons and SNPs [19,20,8]. In parallel, informatic proteome pipelines are also becoming more "genome-centric". Examples include the genome annotating pipeline (GAPP) [21] and PeptideAtlas resources [14,15] which both support the mapping of identified peptides back onto genome viewers [22]. Some experiments have even found these published genomes are annotated incorrectly [23] fully demonstrating the utility of proteome data. These conclusions have sparked interest throughout both proteomics and genomics as to the best ways in which to use this new source of experimental validation of genome annotations [9].

In this project we collected tandem MS data from *A. niger *samples and searched this against predicted protein sequences derived from two independent genome sequences: ATCC1015  by JGI and CBS 513.88 by DSM [24]. The JGI sequence in particular had 87,287 predicted gene models, containing 11,200 "best" models, which we clustered to 8709 genomic loci (Table 1). To generate peptide identifications, tandem MS data was searched against forwards and reversed protein sequence databases derived from the JGI and DSM model sets using Mascot [25]. As well as using standard Mascot scoring, we used a modified version of the Average Peptide Scoring (APS) technique which iteratively calculates peptide filters and reverse database thresholds [26] at various false discovery rates (FDR). By filtering out low scoring peptides using a threshold score this method claims to find more confident protein identifications than the standard Mascot (v2.1) protocol. The APS-identified peptides were then mapped back to the genome via the gene models clustered at each locus. This data offers direct support for predicted open reading frames for two independent *A. niger *genome annotations. For the JGI annotation, the MS data provides support for conflicting gene model predictions and can potentially eliminate inconsistent ones from further consideration. For a significant number of clusters, gene models not hitherto considered the "best" were seen to be more consistent with the experimental data, suggesting they are more likely to be correct or be the principally expressed isoform. This pilot project further demonstrates the utility of proteome data for genome annotation, since it can be used to experimentally validate predicted gene model sets and offer an additional source of evidence that a gene is not only transcribed, but also translated.

**Table 1:** Overview of JGI and DSM *A. niger *genome data

**DOE JGI *A. niger *genome**	
Genome Size	37.1 Mb
Number of Gene Models generated	87,287
Number of filtered "best" Gene Models	11,200
**DSM *A. niger *genome**	

Genome Size	*33.9 Mb*
Number of annotated proteins	14,165

## Methods

### Experimental Aspergillus niger proteomics

#### Aspergillus culture conditions and protein extraction

*Aspergillus niger *strain N402 was cultured in *Aspergillus *media (ACM) at 25°C and 150 rpm. The *A. niger *mycelia (100 mg) were ground down using a pestle and mortar and cells lysed by mechanical glass bead cell lysis. Protein was then extracted using TCA precipitation.

#### 1-D Electrophoresis

*A. niger *extracts were separated on 10%, 12% and 15% SDS-PAGE gels and stained using Coomassie R250. Gel bands were excised from top to bottom of the gel. In-gel tryptic digestion was carried out as described by Shevchenko *et al *[27] and the resulting peptides were extracted by the addition of 2 volumes of Acetronitrile and dried prior to analysis.

#### LC-MS/MS

Liquid Chromatography tandem mass spectrometry (LC-MS/MS) was performed on an UltiMate/Switchos/Famos nanoflow HPLC (*Dionex, Camberley, Surrey*) coupled to a QTof I (*Waters, Manchester*). Prior to analysis, dried samples were redissolved in 6 μl of 0.1% formic acid (v/v). 5 μl of each sample was injected and desalted on a trapping column (*PepMap C18, 300 μm i.d., 5 mm length*) prior to separation on PepMap C18 analytical column (*75 μm i.d., 15 cm length*). Using a 200 nl/min 1 h gradient: 5–90% solvent B (*A *= *2% Acetonitrile, 0.06% formic acid; B = 95% Acetonitrile, 0.05% formic acid, v/v*). Data-dependent switching between MS and MS/MS acquisition was used, with product ion spectra recorded for a maximum of three precursors per cycle.

Peak lists *(.pkl files*) were generated using PeptideAuto in MassLynx 3.4 software (*Waters*), combining all sequential scans for the same precursor, centroiding data with a minimum peak width parameter of 2 and using a peak top parameter of 80%.

### Computational *Aspergillus niger *proteomics

#### Data sources

The main source of data for this project was downloaded from the JGI Genome Portal for *A.niger * and included the genomic scaffolds, the unfiltered set of gene models generated autonomously in the JGI annotation pipeline and also the filtered gene models set. The size of each of these sets is displayed in Table 1 along with statistics for another *A.niger *genome recently sequenced industrially by DSM *Food Specialties ** and *recently published [24].

### Generation of gene models

Gene models in the genome of *Aspergillus niger *were predicted using *ab initio *gene predictor Fgenesh [28] and homology-based methods Fgenesh+ [28] and Genewise [29]. In addition over 15,000 *A.niger *ESTs from GenBank and over 1,200 full-length (FL) mRNAs from RefSeq were directly mapped to genomic sequence and were employed to extend the predicted gene models into FL genes by adding 5' and/or 3' UTRs using the estExt method. Since multiple gene models were generated for each locus, a single representative model from each set of overlapping gene models was selected. This selection was based on homology to proteins from other organisms and available EST support. Gene models overlapping with transposable elements detected in the *A.niger *assembly were excluded from the final set. All these methods are integrated into the JGI annotation pipeline. Finally the initial redundant set of 87,287 gene models predicted by different methods was filtered down to a non-redundant set of 11,200 "best" gene models.

### Mass spectrometry database searching

**Figure 1:**
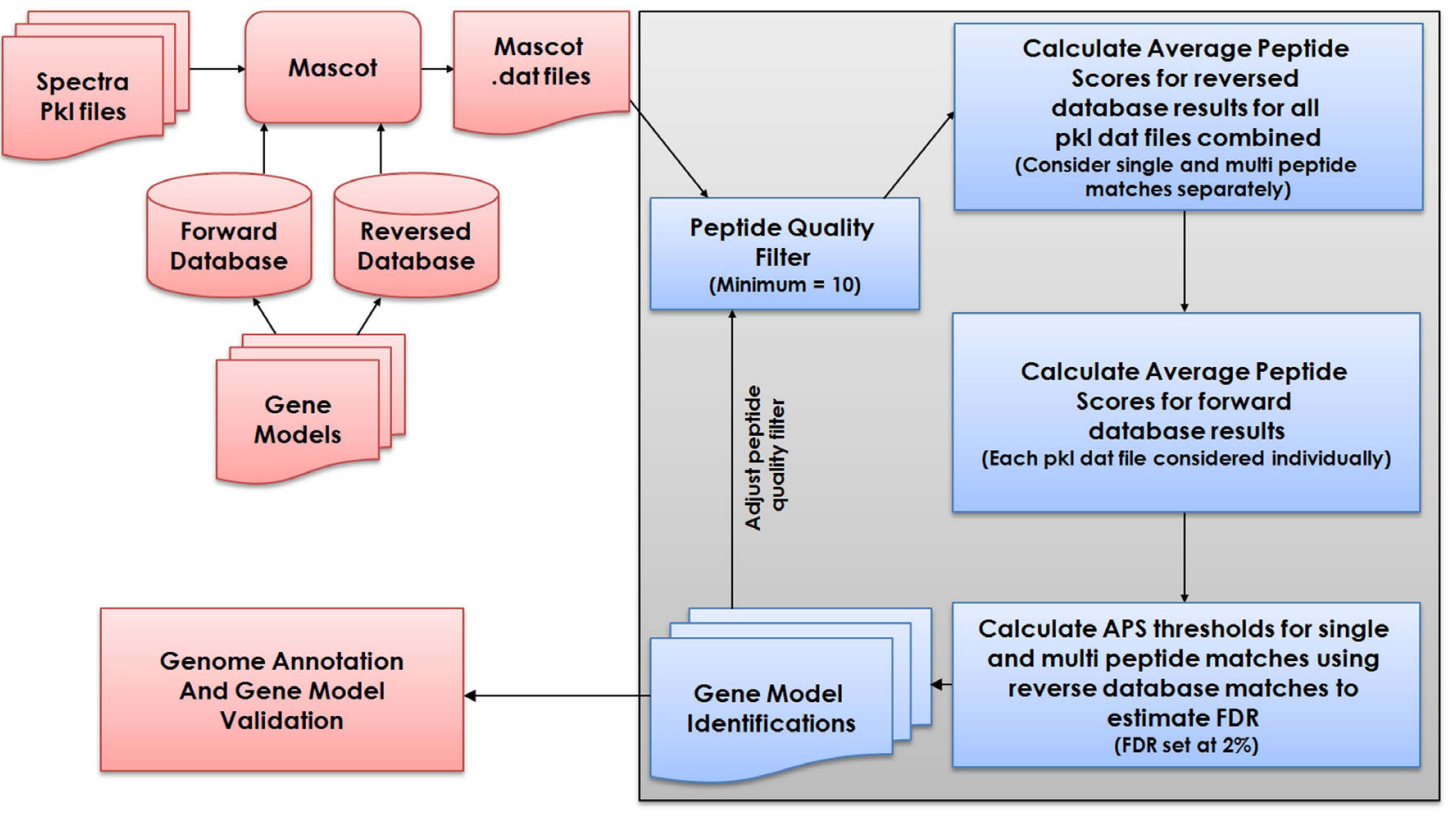
**Schematic of the Average Peptide Scoring (APS) pipeline using reversed database searching**. The final APS threshold is established iteratively for each pkl file searched against a given database, calculated over a range of peptide quality filters. Mascot was used to conduct an initial database search of both forward and reverse databases, and the resulting peptide scores were then used to calculate an average peptide score for matching proteins.

The resulting spectra from the MS analysis of the *A.niger *samples were submitted to a local Mascot server (version 2.1) [25] and searched against both the forwards and reversed versions of each FASTA-formatted protein sequence databank, generated from the JGI and DSM gene model datasets. All Mascot searches used the following parameters: a precursor ion tolerance of 2 Da, fragment ion tolerance of 0.5 Da, a miss cleavage allowance of up to and including 2, and oxidation of methionine residues as a variable modification. Searches were carried out independently for each band cut from the gel. Mascot search results were also further processed using a local implementation of the Average Peptide Scoring (APS) method [26], applying both a peptide quality filter (set to a minimum ion score of 10) and a protein-level APS threshold derived from spectral matches to peptides in a reversed decoy database. (see Figure 1). The APS is calculated as the average peptide score over all putative peptide hits for a candidate protein match. Using a reverse database it is possible to calculate a false discovery rate (FDR) from matches to the forwards and decoy (reverse) database above a given APS protein threshold. The original method implemented by Shadforth and colleagues reported all "forward" protein matches above the maximum APS score which is calculated from the reverse database search results (treating single and multi-peptide protein hits independently). This equates to a theoretical 0% FDR, although this model has its limitations [26,30]. We have made modifications to this approach as it led to some inconsistencies when applied to our dataset of highly redundant protein models and notably where relatively small numbers of spectra were obtained from some gel bands. The latter case led to artificially low maximum Mascot ion and APS scores derived from the reverse database searches, which in turn led to weak identifications being reported. To avoid this, we calculated APS thresholds using the combined reverse database results from all gel bands, again treating single and multi-peptide protein identifications separately. This provides a better background model to estimate chance spectral matches and leads to more conservative APS thresholds. We also applied a more realistic FDR threshold of 2%, calculating false positives from the reverse database hits using the equation below:

$$FDR=\frac{FP}{TP+FP}$$

Where FP is the number of false positive reverse protein hits above the APS threshold, and TP the number of forward protein hits above the threshold less the number of false positives. For example, if 100 forward proteins and 5 reverse proteins exceed the APS threshold, TP = 100 - 5, FP = 5, and FDR = 5/(95+5) = 5%. As the reverse database hits were taken from all searches, the FP values were scaled by the relative number of spectra in each band. The APS protein threshold was lowered until the FDR reached 2%. For each gel band we calculated the optimal peptide score threshold by increasing it from a value of 10 in steps of 1 and calculating the number of APS forward hits at the 2% FDR threshold. The peptide score threshold reporting the largest number of APS hits was used.

All mass spectrometry data and peptide identifications have been deposited with the PRIDE proteomics database , with PRIDE accessions 7972–8124 inclusive.

#### Clustering of the gene models

**Figure 2:**
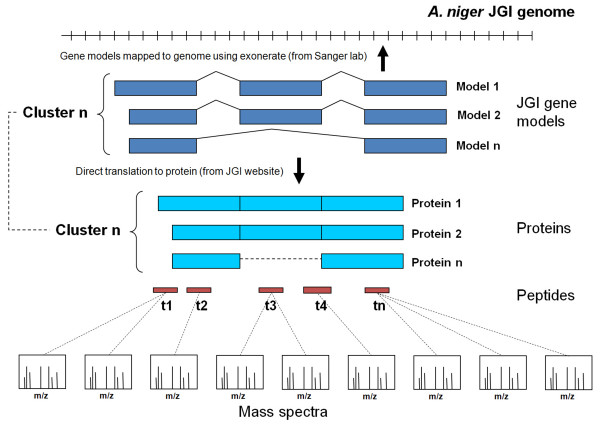
**Overview of the relationship between gene models, clusters and predicted proteins**. An overview of how Aspergillus niger proteomics data is mapped via clusters of gene models, which are in turn mapped back to the genomic scaffold via EXONERATE. This allowed the assessment and evaluation of gene models located at a particular genomic loci based on the peptides consistent with the proposed gene model structures.

At the project outset, complete genomic coordinates were not available for the JGI predicted protein dataset. Hence, as an extra quality control step, all gene models were mapped back to the genomic scaffold using EXONERATE[31] from the European Bioinformatics Institute (EBI) . These were subsequently found to be in agreement with genomic coordinates. Mapped gene models were then grouped into clusters in a simple fashion based on overlapping genomic coordinates (See Figure 2).

#### Analysis of the gene models clusters with proteomics data

For searches conducted against the JGI predicted proteome set from the gene models, each peptide identified as significant using the APS scoring method pipeline was mapped back though the matched gene models to the appropriate genomic scaffold. Gene clusters were then evaluated by ranking the clustered gene models in a tabular format based on the number of peptides matched in the genomic region. Each gene model cluster and affiliated peptides were also visualised using the BioPerl::Graphics module [32]. The peptide data matched and aligned to each cluster of gene models allowed the elimination of inconsistent models from the cluster. This allowed the clusters to be classified into four categories: i) clusters with no matched proteomics data, ii) clusters containing a "best" filtered model which is consistent with all aligned peptides, iii) clusters which do not contain a "best" filtered model but match some proteomics data, and iv) clusters containing a "best" filtered model which is inconsistent with the proteomics data. Categories iii) and iv) are of particular interest since they support novel gene structures not deemed the most likely from the gene prediction pipeline.

## Results and discussion

### Average Peptide Scoring (APS) Results

All 19,628 MS/MS spectra collected from digestions of 153 gel slices from 3 separate runs were searched against the various *A. niger *proteome databases. Spectra collected from individual gel slices were searched against the various protein databases separately, rather than combining spectra from all bands, since peptide identifications common to one slice were more likely to come from the same protein. Table 2 compares the numbers of peptide identifications made using both "standard" Mascot searching and the Average Peptide Scoring (APS) protocol [26]. All peptide, protein and cluster-centric data are available in Additional File 1.

**Table 2:** Protein-level identifications obtained over three search databases.

		**JGI Genes** **All models^a^** **(87,287)**	**JGI Genes** **Filtered models^b ^(11,200)**	**DSM proteins** **(14,165)**
Gel01–12% SDS (partial) 8 bands	APS hits	638	42	40
	Mascot hits(1)^c^	461	38	36
	Mascot hits(2)^d^	448	38	36
				
Gel02–15% SDS (partial) 33 bands	APS hits	1443	109	111
	Mascot hits(1)^c^	1062	102	102
	Mascot hits(2)^d^	1047	102	102
				
Gel03–10% SDS (full) 110 bands	APS hits	2349	153	156
	Mascot hits(1)^c^	1572	146	140
	Mascot hits(2)^d^	1572	146	139

^a^Searches performed against 87,287 protein sequences translated from 87,287 predicted gene models, cluster at 8709 loci on the genome.

^a^Searches performed against the 11,200 "best" gene models, based on homology to known proteins, from the full 87,287 set. Note, some clusters have more than one "best" model, and some have none, hence there are more "best" models than clusters.

^c^Protein hits are reported with 1 or more peptide identification with significant Mascot scores (p < 0.05)

^d^Protein hits are reported with 1 or more peptide identification with significant scores using the Mascot MudPit Score.

The APS search results are compared to two interpretations of direct Mascot searching. Mascot(1) simply reports the number of proteins containing one or more peptides with ion scores above Mascot's default threshold, which estimates significance at p < 0.05 for individual peptides. Mascot(2) results refer to Mascot's MudPit scoring system, the recommended approach when considering large numbers of spectra, which filters out some low scoring peptides. Mascot did not find large numbers of significant protein hits to the reverse databases in this case (typically only 1 or 2 proteins for each experiment). It should be noted that the current version of Mascot (v2.2) also supports reverse database searching directly although we performed equivalent searches here "manually" with the earlier version.

The data presented here provides still further evidence that the APS technique is a simple yet effective strategy to find peptide hits consistent with confident protein-level identifications whilst maintaining a low overall false discovery rate. As noted by Shadforth and colleagues [26], the APS approach removes candidate false positive hits whilst maximising true positive matches by selecting weaker scoring peptides that are consistent with higher scoring peptides in the same proteins. This is broadly equivalent to Mascot's MudPIT scoring system which effectively removes protein hits from multiple low scoring peptide matches.

Table 2 shows results for the two protein sequence databases derived from the JGI genome. The first contained all the automatically generated gene models and the second a reduced set of filtered gene models representing the most likely protein, where appropriate, for each gene locus. The "All Models" database is highly redundant containing 87,287 gene models, and is almost eight times the size of the filtered dataset. This leads to the greater number of APS matches compared to the filtered set; consequently there is also redundancy in these protein matches and the number of hits to the filtered database gives a better reflection of the total number of identified proteins from different gene *loci*. In mitigation, the Mascot significance threshold is dependent on database size and consequently the larger "All Models" database has a higher peptide and protein threshold for reporting significant matches.

The APS scoring method calculates separate thresholds for single and multi peptide identifications [26] and typically for most high throughput proteomics studies, a large proportion of our peptide identifications were "one hit wonders". Although these matches are generally held to be of lesser confidence than multi-peptide matches, other authors have argued against this [33] and the APS methodology does consider them independently with a more stringent threshold (the average multi-peptide APS filter was a Mascot ion score of 34, whereas the single peptide equivalent was 51). Whilst we recognise that the approaches used here are unlikely to completely remove all false positive identifications, we reasoned that a small false positive rate was tolerable for gene model validation where other sources of information may also used. We conducted some further tests to boost confidence in these single peptide identifications, searching them back against the genome sequence using *tblastn *(with no low complexity filtering, and expect value, word size, and database size parameters optimised for short matches). In total, 95.5% of these 149 peptides align to the genome only once (in the original predicted locus), indicating that the majority are indeed unique and not chance matches to another mis-predicted sequence. Although this does not guarantee the Mascot identification of the peptide sequence, this does at least reassure us that given a correct peptide identification, there is no ambiguity in placement on the genome sequence. The few peptides that were unmatched were observed to span introns and could not be matched by using a simple *tblastn *search where the large "gap" was not spanned.

**Figure 3:**
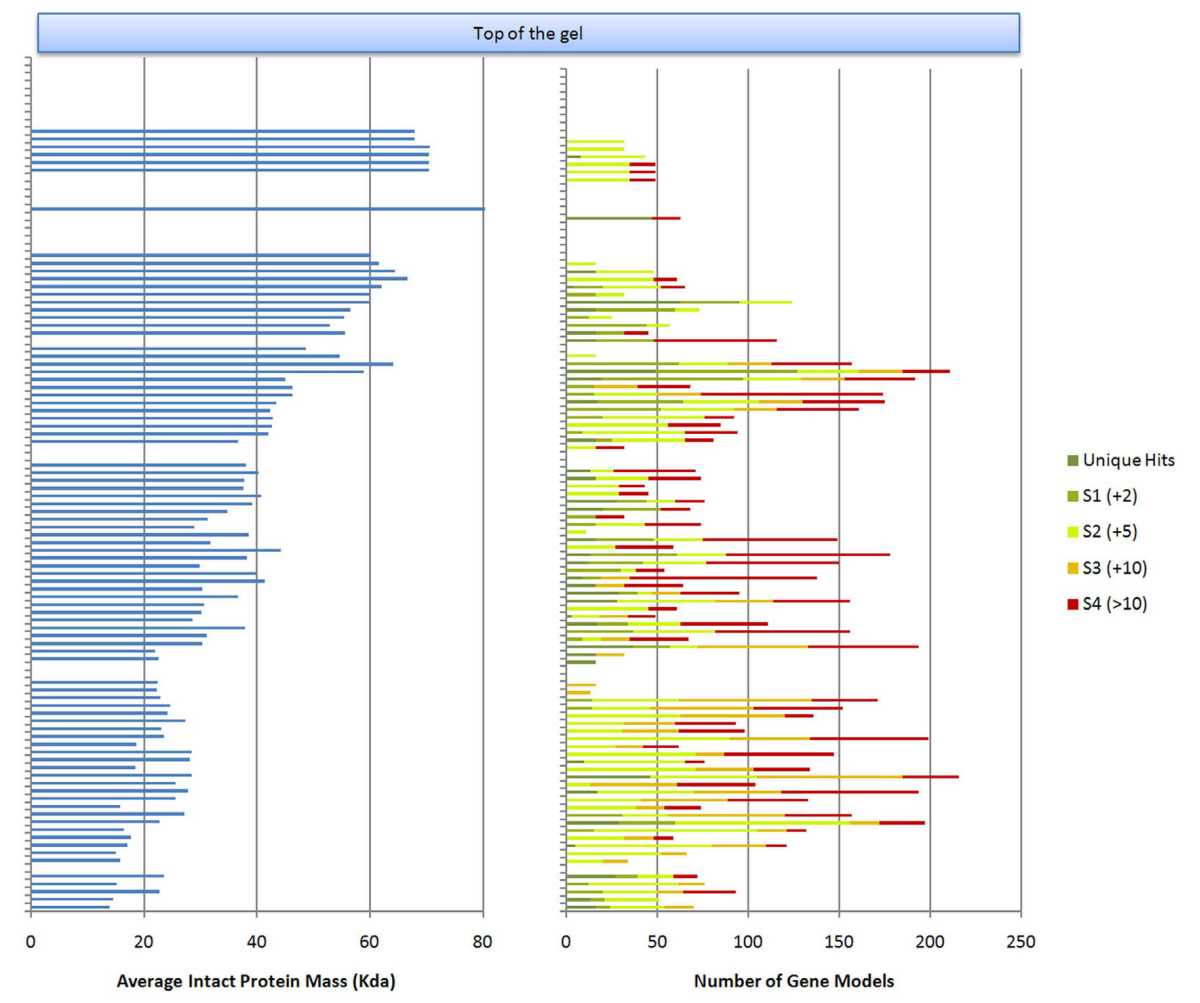
**Gel band protein identifications and their relationship with theoretical molecular weight**. The right hand bar chart shows of the number of protein identifications made from each gel slice using the average peptide scoring method when searching the DSM A. niger database. Dark green sections represent protein identifications unique to that gel band. Subsequent colours, shown in the key at the top of the plot, indicate the distance in consecutive gel bands that a particular set of protein identifications are shared i.e. red shows protein identifications which are found more than 10 bands from the current positions. The left hand bar chart shows the mean protein mass in KDa for proteins identified in each gel band. As should be expected the average protein mass decreases from the top to the bottom of the gel.

We also examined the relationship between the theoretical mass of the matched *A. niger *proteins and gel band position of the protein identifications from the 10% gel. A histogram of protein identifications collated by ordered gel band is compared to an aligned histogram of the average protein mass identified in each band; an example is displayed in Figure 3. As the 1-dimensional SDS-PAGE gel separates proteins based on their mass it is therefore expected that most protein identifications should be localised to a small number of neighbouring bands on the gel. Indeed, over 25% of the identified proteins were unique to a single, specific gel band, with a majority of the identifications shared within a 10 gel band range; indeed most of these were within 5 gel bands. Only a small number of identifications were shared over large distances across the gel. Some of these could be false positive identifications, although it is also possible that truncated gene model predictions, paralogues, alternative isoforms or post-translational modifications could lead to different gel migration properties for some proteins. For example, closely related protein homologues in the same gene family will be indistinguishable from each other if only a small number of common peptide identifications are available.

Overall, these results are generally reassuring since the proteins used here represent a range of predicted gene models and there is a clear trend in decreasing protein mass down the gel. A high false positive rate would lead to many protein identifications outside of the expected mass range, decreasing the correlation with average mass shown in Figure 3. A simple correlation of average protein mass against gel band number results in a Pearson correlation of 0.94, which supports this. This is considerably higher than would be expected by chance (p < 10^-20^) with a mean correlation of 0.01 (s.d. 0.1) obtained by shuffling the protein masses in a simulation. Similar results are obtained when searching against either the JGI filtered gene set or DSM gene set (data not shown) suggesting there is no bias produced by a given gene set.

### Mapping of proteomics data to gene model clusters

The complete set of 87,297 JGI *A.niger *gene models were mapped back to the genome using EXONERATE to generate 8709 overlapping clusters, some of which span multiple genes. The clusters were then filtered, limiting the set to those which contained peptide mappings from the proteomics data. These 214 clusters contained 4093 individual gene models of which 2872 were supported by matched peptide data. As can be seen in Table 3, the 214 models were further classified into 3 categories. The first and most prevalent were those clusters containing a model from the JGI filtered "best" dataset which was itself supported by the proteomics experimental evidence. The second category contained proteomic evidence, but no model from the filtered "best" dataset mapped to that genomic locus – sufficient evidence to assign a preferred gene model was not available at this locus. Finally, the third category contained clusters that did contain a filtered "best" model but this model was unsupported by the proteomic data whereas an alternative, conflicting, model was. This suggests an alternative gene structure for this locus is more likely. The first category provides experimental validation that the "best" gene model in that cluster is the most representative at the given genomic locus and if nothing else increases confidence in those models. The later two categories covered 13 of the JGI clusters, each one suggesting either an addition or alteration to the filtered "best" model dataset. In total this represents over 6% of all gene clusters with accompanying proteomic data. However, even though the proteomics data boosts confidence in individual gene models and suggests alterations to the filtered set of "best" models, the total peptide coverage on many of these gene models is often poor. This does not therefore provide conclusive validation of a particular gene model over another; rather it offers additional support at the protein level that a particular isoform is expressed from that particular genomic locus. This is analogous to the use of ESTs/cDNAs to lend weight to gene predictions; absence of transcriptional data does not in itself disprove a candidate gene model, but when direct physical evidence of a different gene structure at the same, over-lapping, locus is observed it reduces the confidence that it is correct or the principal expressed isoform in those experimental conditions.

**Table 3:** Gene cluster and proteome peptide identification results.

	Number of APS matches (to Gene clusters, Proteins, or Peptides)	i) "Best" filtered model consistent with peptide data	ii) Gene cluster does not contain a "Best" filtered model, but does have APS matches	iii) "Best" filtered model in cluster is inconsistent with peptide data
Gene Clusters	214	201	9	4
*Protein identifications*				
Total	2872	2729	56	87
Single peptide	1791	1698	28	65
Multi-peptide	1081	1031	28	22
*Peptide identifications*				
Unique	405	379	13	13
(single peptide hits)	(149)			
(multi-peptide hits)	(256)			

(Identified peptides validating exon/intron boundaries: 54 peptides covering 54 introns)

Of the 8709 gene model clusters generated only 214 contained individual gene models with significant APS scores. These 214 clusters were then classified into three distinct types; a). those where all peptide identifications were consistent with the filtered gene model selected as the best model for that particular genomic locus, b). those where the cluster did not include a "best" filtered model but still contained models with matching peptide identifications, and c). those clusters where the "best" filtered model either did not match or other models in the cluster were more parsimonious with the proteomics data suggesting that the filtered model was not the most likely model for that particular gene locus.

Putting the peptide identification data in to the context of our results, we examined the 4093 models in the 214 gene clusters with associated APS peptide matches. Of these, 1221 of the models were classified as inconsistent with the proteomic data, which represents about 30% of them. This is, on average around 5 models per cluster; clusters contain 16 models on average.

One of the most informative types of peptide identification involves those that spanned an intron/exon boundary. We matched 54 peptides spanning introns. These identifications provide proteomics evidence for predicted splice sites in the gene models above and beyond any available transcript data or orthologues.

### Further analysis of gene model clusters

We analysed the 13 unusual JGI clusters in more detail, additionally comparing them with the genes predicted in the DSM genome sequence, and reporting BLAST matches from the UniRef90 database. Results are shown in Table 4. In all but one case, a homologous gene is predicted in the DSM annotation and a UniProt homologue is detected with a highly significant e-value of 10^-30 ^or better in another species. This lends weight and consistency to their validity as genuine protein coding genes.

**Table 4:** Gene cluster statistics where "best" filtered model is inconsistent with proteome peptide data

**Cluster ID**	**DSM E-Value**	**DSM Protein**	**DSM Protein Description**	**UniRef** **E Value**	**UniRef Protein**	**UniRef Protein Description**
68_S6	2E-64	An15g00690	strong similarity to 14.8 kD subunit of NADH:ubiquinone reductase – Neurospora crassa	2E-54	Q1E404	Hypothetical protein; n = 1; Coccidioides immitis RS|Rep: Hypothetical protein – Coccidioides immitis RS
229_S11	5E-41	An09g03480	strong similarity to snRNA-associated sm-like protein Lsm2 – Saccharomyces cerevisiae	8E-35	Q1E7Q6	Hypothetical protein; n = 1; Coccidioides immitis RS|Rep: Hypothetical protein – Coccidioides immitis RS
626_S3	4E-71	An08g03600	similarity to hypothetical protein CAE47874.1/AfA24A6.130c – Aspergillus fumigatus	3E-47	Q0D146	Predicted protein; n = 1; Aspergillus terreus NIH2624|Rep: Predicted protein – Aspergillus terreus NIH2624
25_S5	0	An07g09990	strong similarity to heat shock protein 70 hsp70 – Ajellomyces capsulatus [putative frameshift]	0	Q56G95	Heat shock protein 70; n = 2; mitosporic Trichocomaceae|Rep: Heat shock protein 70 – Penicillium marneffei
523_S5	1E-75	An07g01640	strong similarity to calmodulin 6 CaM6 – Arabidopsis thaliana	7E-70	Q4WGR4	EF-hand protein; n = 2; Aspergillus|Rep: EF-hand protein – Aspergillus fumigatus (Sartorya fumigata)
303_S2	1E-37	An02g05240	strong similarity to histone 4 from patent WO9919502-A1 – Homo sapiens	8E-38	UPI00005A5829	PREDICTED: similar to germinal histone H4 gene; n = 1; Canis familiaris|Rep: PREDICTED: similar to germinal histone H4 gene – Canis familiaris
117_S3	2E-131	An06g00990	strong similarity to soluble cytoplasmic fumarate reductase YEL047c – Saccharomyces cerevisiae	2E-71	Q0CC76	Hypothetical protein; n = 1; Aspergillus terreus NIH2624|Rep: Hypothetical protein – Aspergillus terreus NIH2624
54_S17	9E-139	An04g06870	similarity to hypothetical protein CAD21072.1 – Neurospora crassa	2E-121	Q4WPR6	Transcription factor RfeF, putative; n = 1; Aspergillus fumigatus|Rep: Transcription factor RfeF, putative – Aspergillus fumigatus (Sartorya fumigata)
717_S2	0	An02g11680	strong similarity to translation initiation factor eIF-4A – Schizosaccharomyces pombe	0	Q5B948	ATP-dependent RNA helicase eIF4A; n = 1; Emericella nidulans|Rep: ATP-dependent RNA helicase eIF4A – Emericella nidulans (Aspergillus nidulans)
39_S9	5E-72	An12g09130	similarity to glucanase ZmGnsN3 from patent WO200073470-A2 – Zea mays	7E-130	Q4WCP3	Hypothetical protein; n = 1; Aspergillus fumigatus|Rep: Hypothetical protein – Aspergillus fumigatus (Sartorya fumigata)
373_S9	No Match			0	Q2TZ90	Ca2+binding actin-bundling protein; n = 2; Aspergillus|Rep: Ca2+binding actin-bundling protein – Aspergillus oryzae
256_S9	3E-125	An12g04870	strong similarity to cytoplasmic ribosomal protein of the large subunit L10 – Saccharomyces cerevisiae	1E-117	Q2TZP5	RIB40 genomic DNA, SC011; n = 2; Aspergillus|Rep: RIB40 genomic DNA, SC011 – Aspergillus oryzae
238_S10	2E-124	An18g04220	strong similarity to mitochondrial ADP/ATP carrier anc1p – Schizosaccharomyces pombe	4E-115	Q4WJN2	Mitochondrial ADP, ATP carrier protein (Ant), putative; n = 1; Aspergillus fumigatus|Rep: Mitochondrial ADP, ATP carrier protein (Ant), putative – Aspergillus fumigatus (Sartorya fumigata)

**Figure 4:**
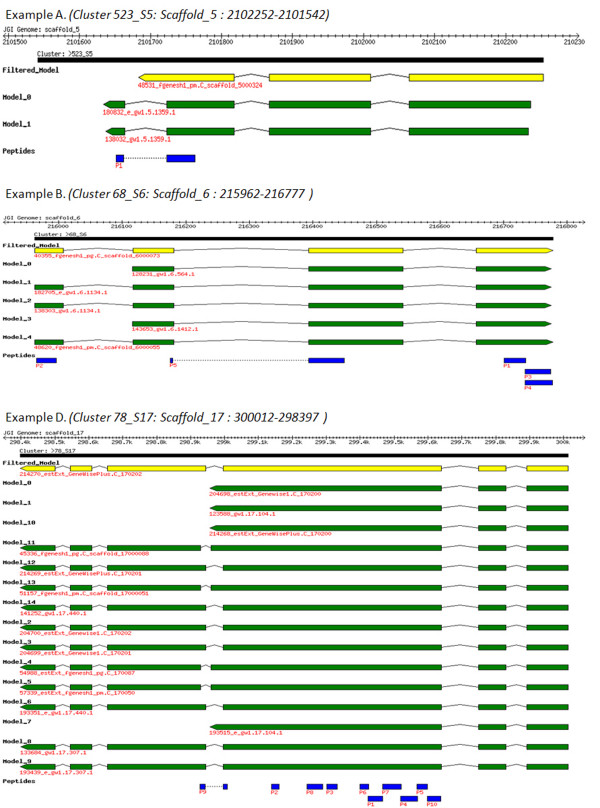
**Example Gene Clusters with associated peptide identifications**. Three example mappings of proteomics data to clusters via the gene models are shown. The images, generated using the BioPerl::Graphics module, are split into tracks with the top ruler representing the genomic scaffold and the first track coloured black highlighting the region covered by the cluster. The tracks below this represent all the gene models mapped to this cluster regardless of whether they have proteomics evidence or not. The yellow models correspond to those that are included in the filtered "best" gene model set; models not in the filtered set are coloured green. The final and bottommost track represents the peptides as mapped to the genome track and are coloured blue. Each example shows how the proteomics evidence in the form of significantly matched peptides can lend weight to support or refute the filtered gene model set.

**Figure 5:**
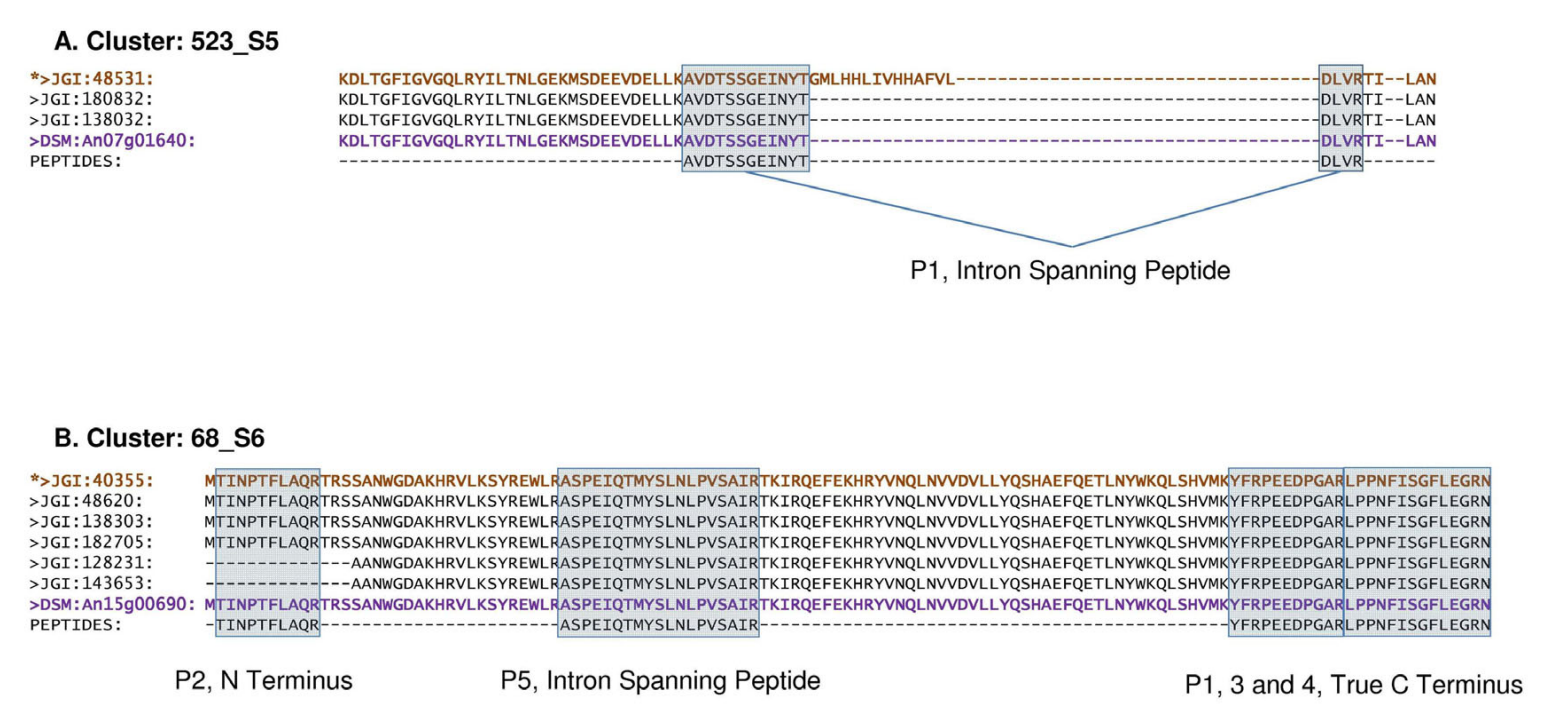
**Example gene cluster alignments**. Selected sections from the examples in Figure 4 are shown as alignments illustrating peptide mappings to the gene models. Peptide identifications are shown in shaded boxes, corresponding to those in Figure 4, with intron spanning peptides linked by a solid line. The "best" filtered models are shown in bold text, the corresponding aligned DSM proteins in purple, and gene models consistent with the proteomics data are preceded by an asterisk.

We also generated a visual representation of each of the 214 clusters using the BioPerl::Graphics module, with each gene model and its significant peptides aligned to the genomic scaffold. This allowed us to manually investigate each cluster to see exactly how our proteomics evidence supports certain gene model structures over others. Figure 4 contains 4 example clusters with associated peptide identifications and illustrates the cluster redundancy with considerable similarity in the different gene models predicted from the various pipelines. The examples shown in Figure 4 are typical with similar exonic structure throughout; most of the variation between gene models occurs in the terminal regions. Cluster 523_S5: scaffold_5:2102252-2101542 (*example A*, Figure 4) maps a single peptide to a cluster of just three gene models. In this case, the gene predictions have identified a "best" filtered model (shown in yellow) as well as two further candidates. The proteomics data offers direct positive evidence that the two non filtered models are more likely. This is due to the peptide spanning a predicted intron at the C-terminus of the models, which is absent in the filtered model. This cluster represents a Calmodulin protein from sequence similarity searches against UniRef90 and DSM databases. The P1 peptide, AVDTSSGEINYTDLVR, which distinguishes the predicted JGI models, is also consistent with the DSM predicted protein as shown in Figure 5a. All alternative models including the filtered model read through this section of the genome and do not contain an intron.

The second example, cluster 68_S6: Scaffold_6: 215962–216777 shows 5 peptides clearly supporting the filtered model and validating one of the three introns in the gene models. These gene modes show strong similarity to ubiquinone reductase in other filamentous fungi. Three of the peptides are clustered close together at the C-terminus and one at the N-terminus, two areas of the gene models that can be very difficult to validate and correctly predict. Figure 5 shows a more detailed look at how the peptides clearly match the terminal regions of the predicted gene models.

Finally, example cluster 78_S17:scaffold_17:300012-298397, which shows similarity to Aspartate aminotransferase, does contain a "best" filtered model which matches all the peptide data. The models in the cluster are similar with all the predicted structural differences occurring around one particular intron which is validated by one of the peptides. This cluster is supported by a large number of proteome peptides which gives good coverage of the models validating the key intron where the predictions differ. These examples provide a clear demonstration of how proteomic evidence can support and refine gene models predicted from sequenced genomes, lend weight to predictions and help reconcile different potential gene structures in the annotation process.

### Comparison of DSM and JGI genomes

Another strain of *A. niger *has also been recently sequenced and published by DSM [22] and we compared the collected proteome data here using Mascot searches against both the DSM and JGI predicted protein sets, shown in Table 2 and Figure 3. Using a simple reciprocal top-hit BLAST approach we defined equivalence relationships between the DSM proteins and the JGI gene models. As would be expected, both protein datasets have a proportion of proteins with no similarity in the other database, and we have proteomics evidence for some of these unique proteins. In fact 9 DSM proteins had significant APS peptide identifications but had no corresponding gene model in the JGI dataset and, *vice-versa*, 130 JGI gene models (corresponding to 18 distinct clusters) with proteomic data had no equivalent protein in the DSM database. This suggests that some possible gene models have not yet been generated for the JGI genome which would fit into the "best" filtered dataset and also that several proteins have been missed in the DSM annotation which are included in the JGI gene models set.

## Conclusion

Proteomics would not be possible without genomics; however, this does not mean that it is powerless to assist genomics. In fact quite the contrary, proteomics provides a fast, relatively cheap and confident method for gathering a large amount of experimental evidence to assist genome annotation. It also has the added advantage of confirming that transcripts are translated to the proteome stage and can help identify functional details of the mature protein form which includes N- and C-termini and post-translational modifications. Here we present a relatively modest scale study on a fungal organism of current interest and hence our data has a relatively limited coverage of the entire *A. niger *proteome. Recent publications point out the need to conduct multiple high throughput experiments under a variety of conditions to achieve complete proteome coverage [34] and we have only used one here. Despite this, and using a "hot off the press" genome annotation, we were able to offer proteomic support for 214 genes with proteomic data offering refinements to predicted gene models in around 6% of these cases. These represent 13 gene predictions for which there was uncertainty in the annotation (no filtered model) or were potentially incorrectly annotated in the original gene model selection process. Importantly, as some of the examples in Figure 4 highlight, there is often uncertainty surrounding the true N- and C-termini of genes and most of the variability in the gene model structures exists at these regions. We were able to offer concrete data to help resolve some of these ambiguities in a number of cases, but it should be noted that we have not employed a targeted strategy to protein termini here. One attractive potential solution would be to use an N-terminal specific peptide preparation to enrich for the peptides [35].

We believe that this feedback of experimental data into genomic annotation pipelines could well be formalised and assist in gene prediction pipelines, as has been recently been demonstrated for *Arabidopsis *[36]. Proteomics has the advantage of providing direct evidence for gene products rather than any intermediate stage in the transcription process, and the field has been slow to incorporate proteomics data formally into gene prediction models. However, we hope that this work and others offers strong support for its inclusion, using MS-based peptide identifications as a similar line of evidence to ESTs.

A further point of caution is also necessary. In *Aspergillus*, the level of alternative splicing that occurs has yet to be fully characterised, but appears to be modest [37]. In most cases, the choice for gene prediction is therefore which of the candidate models is most consistent with the data and most likely to be correct. For species exhibiting higher levels of splicing, with multiple isoforms from a single locus, it is more challenging to interpret the data. Multiple peptide identifications could belong to two or more isoforms and it is quite possible that several isoforms are present in the same sample, especially when multiple tissues are studied.

One final caveat that concerns many in the proteomics field is the "one hit wonder" syndrome [33] – proteins with only a single confident peptide identification. Although the majority of our peptide identifications were not in this category, they are still more likely to be false positives. Using the APS approach with independent thresholds for single peptide matches attempts to put them on a consistent protein level confidence to the multi-peptide hits. Indeed, the APS threshold was higher for single peptide hits (51 compared to 34 on average). However, more work is clearly needed to convince practioners of their validity, but appropriately weighted and considered they can offer genuine experimental evidence to support gene models as we have demonstrated here.

## Authors' contributions

JW carried out all the proteomic informatic data analysis, DS, SF-M, IR-G obtained the samples, ran gels and performed all preparations and mass spectrometry, IVG and SEB calculated and supplied gene models and protein predictions. RJB, SJG participated in the overall design of the experimental work. The overall design including all the informatics was carried out by SJH. JW and SJH wrote the paper with contributions from all authors. All authors read and approved the final manuscript.

## Supplementary Material

Additional File 1 **Proteomic peptide identification data from the APS analysis against the JGI gene models.** Lists of all peptide identifications for the APS searches against the JGI predicted gene models are provided, in three different styles. Three worksheets present the data either from the peptide, protein or gene cluster viewpoint. Click here for file
